# Evaluation of appropriateness of second-generation 320-row computed tomography for coronary artery disease

**DOI:** 10.1186/s40064-015-0866-1

**Published:** 2015-03-05

**Authors:** Daisuke Utsunomiya, Seitaro Oda, Hideaki Yuki, Megumi Yamamuro, Kenichi Tsujita, Yoshinori Funama, Morikatsu Yoshida, Masafumi Kidoh, Hisao Ogawa, Yasuyuki Yamashita

**Affiliations:** Diagnostic Radiology, Faculty of Life Sciences, Kumamoto University, 1-1-1, Honjo, 860-8556 Kumamoto-shi, Kumamoto, Chuo-ku Japan; Cardiovascular Medicine, Faculty of Life Sciences, Kumamoto University, Kumamoto, Japan; Medical Physics, Faculty of Life Sciences, Kumamoto University, Kumamoto, Japan

**Keywords:** Cardiac, CT-Angiography, Heart, Appropriate use criteria

## Abstract

The influence of newer-generation CT on the clinical indications and appropriateness of cardiac CT has not been adequately surveyed. We aimed to evaluate the distribution of appropriateness ratings and test the outcomes of cardiac CT using second-generation 320-row CT. The 2010 appropriate use criteria (AUC) were applied at the point of service to a consecutive series of patients (N = 309) who were referred for cardiac CT. The CT indication was determined based on interviews and medical records. The proportions of patients within the categories of appropriate (A), uncertain (U), inappropriate (I), and not covered were described. The prevalence of significant coronary artery disease (CAD) was also compared among the categories. The proportions were 49.2%, 25.9%, and 20.7% for appropriate, uncertain, and inappropriate indication, respectively. The indication that was not covered was only 4.2%. Significant CAD was more frequently observed for uncertain- than appropriate indication (42.5% vs 27.6%; P = 0.03), although the number of significant stenosed segments was not different (P = 0.13). The recent advancement of cardiac CT increased the proportion of uncertain scans, which were associated with a high prevalence of significant CAD.

## Introduction

The recent development of 64-row or more CT provides high-image quality and diagnostic accuracy for cardiac CT, leading to increasing applications for patients with suspected coronary artery disease (CAD). The appropriate use criteria (AUC), developed by the American College of Cardiology and others, provide guidance regarding the appropriateness of cardiac CT for any given clinical situation (Hendel et al. 2006; Taylor et al. 2010). These criteria were developed through a formal process in which expert opinion, guided by the available evidence. The different scenarios are designed based on practical experience. They are tested against clinical trials, reviewed by experts, and rated for their appropriateness into 3 categories of appropriate (A), uncertain (U), and inappropriate (I) (Rich et al. 2012). The first AUC for cardiac CT were published in 2006, followed by an updated version in 2010, including a more complete classification of the conditions and a large shift in appropriate rating (Rich et al. 2012; Taylor et al. 2010; Cullen et al. 2013). Among the 93 cardiac indications in the 2010 AUC, 35 were classified as appropriate, and 58 were judged either inappropriate or uncertain (Taylor et al. 2010).

Cardiac CT has undergone further technical advancement with improved image quality and safety (Leipsic et al. 2012; Nakaura et al. 2013; Oda et al. 2014; Rich et al. 2012). The combination of prospective ECG-gated axial scan and iterative reconstruction technique drastically reduces the radiation- and contrast material dose (Rich et al. 2012; Tatsugami et al. 2012). In 2013, a second-generation 320-row CT scanner was introduced in clinical practice (Chen et al. 2013). Reportedly, the faster rotation time (0.275 s), wider volume coverage, iterative reconstruction, automated exposure control, and larger X-ray power generator provide excellent image quality over a wide range of body sizes and heart rates at low radiation dose (Chen et al. 2013; Khan et al. 2011). However, the 2010 AUC for cardiac CT were basically established on the use of 64-row CT. The impact of the newer-generation CT with more than 64-row detectors on the AUC rating of clinical indications has not been adequately investigated. We hypothesized that the less-invasive scanning and improved image quality by the newer CT could change and widen the cardiac CT indications and appropriateness.

Thus, the purpose of our survey was to assess the distribution of appropriateness ratings and test outcomes in patients referred for less-invasive cardiac CT using the second-generation 320-row CT.

## Methods

### Ethics statement

The study conforms to the principles of the Declaration of Helsinki and approved by the Ethics Committee of the Faculty of Life Sciences, Kumamoto University, and written informed consent was obtained from all patients. This study was registered at the Protocol Registration System of ClinicalTrials.gov (NCT02033837).

### Study population

Between January-01- and December–31–2013, we prospectively applied the 2010 AUC at the point of service to 345 consecutive patients who were referred to our hospital for cardiac CT. Patients were excluded from analysis if there was insufficient clinical information available (N = 6), or if they underwent cardiac CT for the evaluation of aortic disease, pulmonary embolism, or pulmonary vein anatomy because these conditions are not directly related to CAD (N = 30). Therefore, a total of 309 patients were enrolled in this analysis.

### Data acquisition

All patients underwent cardiac CT on a 320-detector CT scanner (Aquilion ONE ViSION Edition, Toshiba Medical Systems, Otawara, Japan). The acquisition parameters were: 240, 280, or 320 × 0.5-mm detector collimation, 275-ms tube rotation time, 270–800 mAs tube current-time product with automatic exposure control (noise index, 20), and 80-, 100-, or 120-kVp tube voltage, according to the patient body mass index (BMI) (80 kVp for BMI < 21; 100 kVp for BMI 21–25; 120 kVp for BMI >25). The CT images were reconstructed using the adaptive iterative reconstruction technique (AIDR 3D, Toshiba Medical Systems, Otawara, Japan).

### Cardiac CT appropriateness

The patients were interviewed, and the medical records were reviewed to customize the cardiac CT indication. Pretest CAD probability and coronary heart disease risk were estimated using the published AUC methods. Two radiologists (10 and 6 years of experience in cardiac CT) independently classified the AUC rating of each patient as appropriate, uncertain, inappropriate or not classifiable. The presence and number of coronary segments with significant CAD were also evaluated. Two experienced radiologists consensually interpreted the coronary CT angiogram including axial source-, curved multiplanar reformation images, and angiographic view. Significant CAD was defined as >50% stenosis. We also recorded the machine-generated volume CT dose index (CTDIvol) (mGy) per examination for imaging during cardiac CT. The CTDIvol averages the radiation dose over the center slice of a CT study comprising multiple parallel slices. The data acquisition range and the dose-length product were calculated on the basis of CTDIvol and the data acquisition range. Finally, the effective radiation dose of the chest was calculated with the equation: effective dose = dose-length product × 0.014 (Hausleiter et al. 2009).

### Statistical analyses

Numerical data were expressed as the mean ± standard deviation. The proportion of scans within the appropriate, uncertain, or inappropriate categories were compared with the 2010 AUC ratings using the Chi-square test. The number of vessel segments with >50% stenosis in the three categories was compared using the Tukey–Kramer test. All analyses were performed using the statistical software JMP 9.0.2 (SAS Institute, Cary, NC). P-values of <0.05 were considered statistically significant.

## Results

### Demographic variables

The patients had a mean age of 67 years (range: 29–89 years), and generally presented symptoms of hypertension and/or hyperlipidemia (Table 1). But they rarely had a history of myocardial infarction, prior percutaneous coronary intervention, or a coronary artery bypass graft (CABG). Prospective ECG-gated 1-beat and 2- or 3-beat axial scanning was performed on 241 and 42 patients, respectively. Retrospective ECG-gated helical scan was performed on 26 patients for CABG assessment. The average effective radiation dose was 1.8 ± 0.4 mSv, 5.5 ± 1.4 mSv, and 18.1 ± 3.6 mSv for 1-beat-, 2-beat-, or 3-beat axial and helical scanning, respectively. Mean contrast material volume used was 43.2 ± 15.9 mL.

**Table 1:** **Demographic variables of 309 patients referred for cardiac CT**

**Characteristics**	
Males	176 (57.0%)
Age	67.3 ± 12.3 years
Hypertension, N (%)	210 (68.0%)
Hyperlipidemia, N (%)	148 (47.9%)
Diabetes, N (%)	74 (23.9%)
Smoking history, N (%)	Prior: 70 (22.7%)
	Current: 61 (19.7%)
Family history, N (%)	160 (51.8%)
History of myocardial infarction, N (%)	49 (15.9%)
Prior PCI and/or CABG, N (%)	38 (12.3%)
Tube voltage, N (%)	80 kVp: 24 (7.8%)
	100 kVp: 185 (59.9%)
	120 kVp: 100 (32.3%)
Median radiation dose	1.9 mSv
	(interquartile range, 1.5–2.6 mSv)

Note _ CABG, coronary artery bypass graft; PCI, percutaneous coronary intervention.

### Distribution of appropriateness of cardiac CT

**Figure 1:**
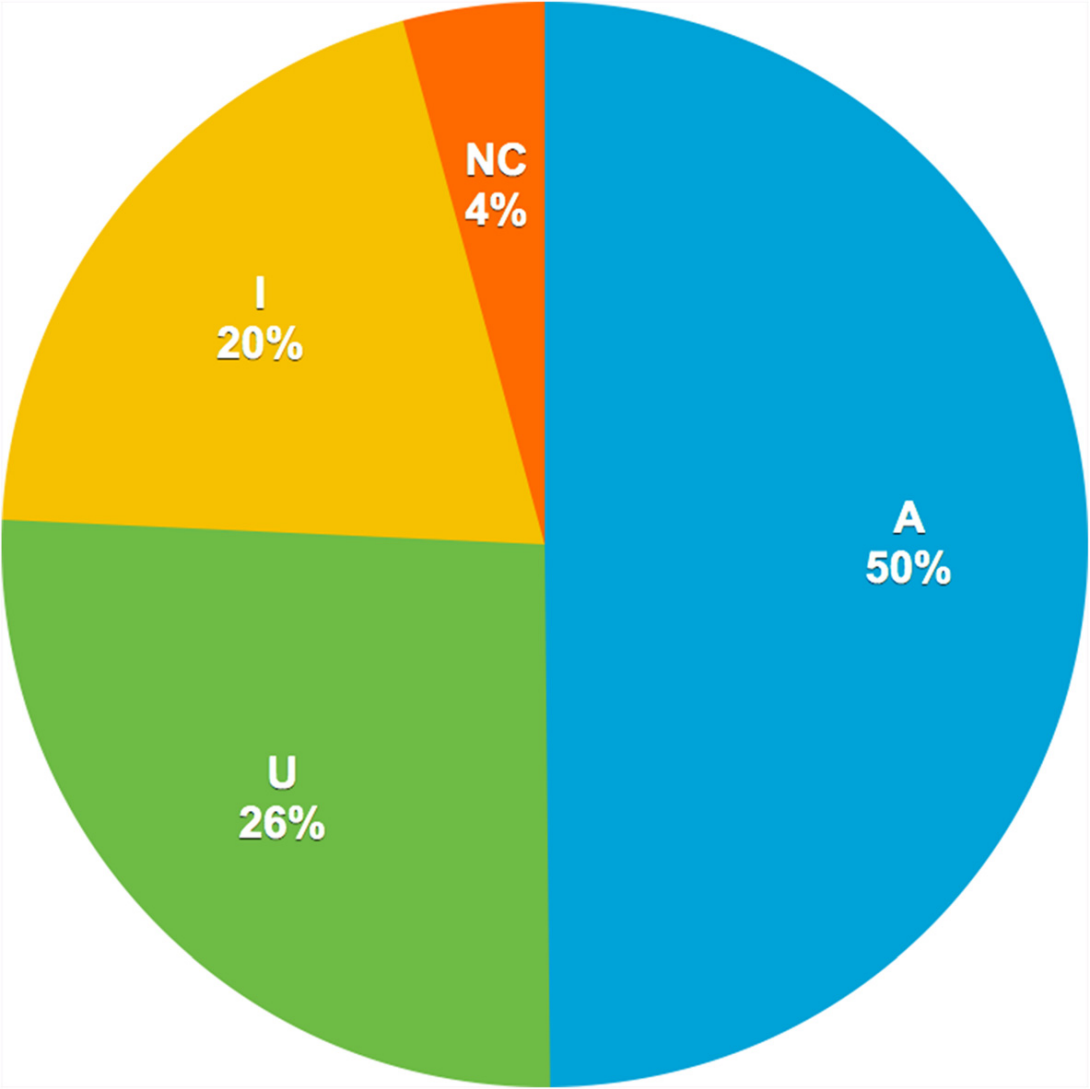
**Distribution of patients classified according to the 2010 appropriate use criteria.** Note _ A, appropriate; U, uncertain; I, inappropriate; NC, not classifiable.

**Figure 2:**
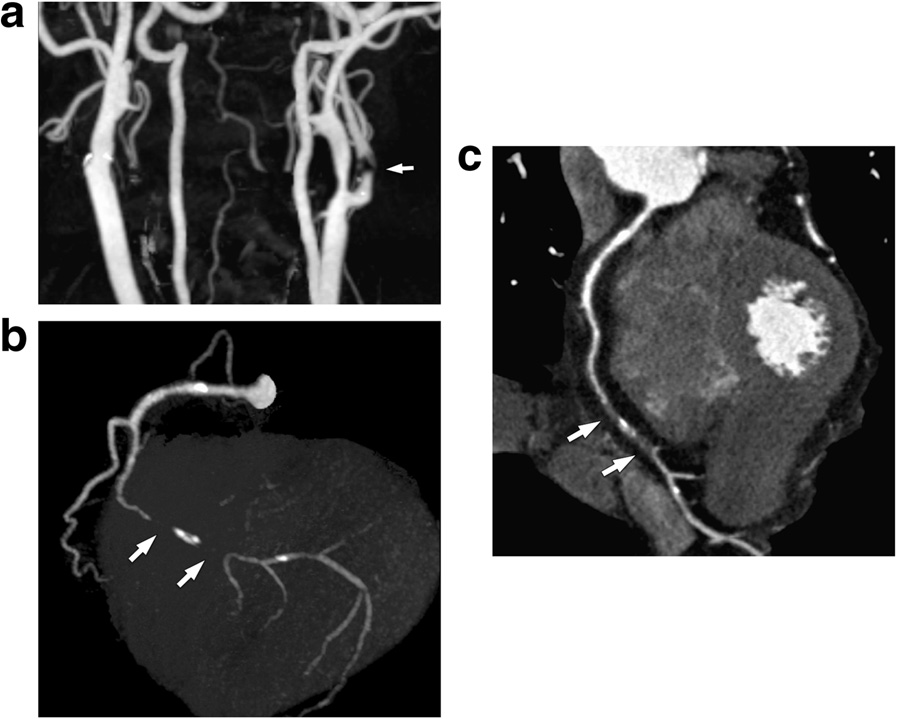
**Carotid CT- (a) and coronary CT angiograms (b and c) of a 70-year-old asymptomatic male undergoing carotid endarterectomy.** Carotid CT angiogram **(a)** shows severe stenosis of the left internal carotid artery. For screening of coexisting coronary artery disease, he underwent coronary CT angiogram prior to carotid endarterectomy. Coronary CT angiographic images **(b)**: maximum intensity projection image [angiographic view], **(c)**: curved planar reformation image) show a long mixed plaque with severe luminal stenosis in the distal right coronary artery.

One hundred and fifty-two of 309 (50%) patients were classified into appropriate indication, whereas 80 (26%) and 64 (20%) patients were classified into uncertain and inappropriate indications, respectively (Figure 1). Only 13 scans (4%) could not be classified. The most common category of cardiac CT indications (29.8%) was the detection of CAD in symptomatic patients without known heart disease (Table 2). Four other indications accounted for 12%–16% of the patients, including CAD detection in other scenarios, and preoperative risk assessment of non-cardiac surgery in the absence of active cardiac conditions. In the latter case, the cardiac CT scan revealed significant coronary arterial stenosis in 20 of 46 patients (43.5%). A representative case for preoperative risk assessment of non-cardiac surgery is shown in Figure 2a, b and c. The scenarios were rated according to the 2010 AUC system, and the top four indications were rated as appropriate (Table 3). The 5^th^ most frequent indication (5.2%) was global coronary heart disease risk estimate in asymptomatic/no known CAD patients with intermediate pretest probability, which was rated as inappropriate by AUC 2010.

**Table 2:** **Category of cardiac CT indications under 2010 appropriate use criteria**

**Category**	**N (%)**
Detection of CAD in symptomatic patients without known heart disease	92 (29.8%)
Detection of CAD/risk assessment	18 (5.8%)
Detection of CAD in other clinical scenarios	48 (15.5%)
Use of CTA in the setting of prior test results	39 (12.6%)
Risk assessment preoperative evaluation of non-cardiac surgery without acute cardiac condition	46 (14.9%)
Risk assessment post revascularization (PCI or CABG)	38 (12.3%)
Evaluation of cardiac structure and function	15 (4.9%)
Not classifiable	13 (4.2%)

Note _CABG, coronary artery bypass graft; CAD, coronary artery disease; CTA, computed tomography angiography; PCI, percutaneous coronary intervention.

**Table 3:** **Most common indications under 2010 appropriate use criteria**

**2010 AUC: most frequent indications**	
Non-acute symptoms possibly representing an ischemic equivalent– interpretable ECG AND able to exercise–intermediate pretest probability (A)	45 (14.6%)
Reduced left ventricular ejection fraction–low or intermediate pretest probability (A)	25 (8.1%)
CTA after prior stress imaging procedure deemed equivocal (A)	18 (5.8%)
Non-acute symptoms possibly representing an ischemic equivalent–uninterpretable ECG OR unable to exercise (A)	18 (5.8%)
Global CHD risk estimate–asymptomatic/no known CAD–low or intermediate pretest probability (I)	16 (5.2%)

Note _ A, appropriate; AUC, appropriate use criteria; CAD, coronary artery disease; CHD, coronary heart disease; CTA, computed tomography angiography; ECG, electrocardiogram; I, inappropriate; PCI, prior percutaneous coronary intervention; U, uncertain.

### Relationship of CAD and appropriateness at cardiac CT

Significant coronary arterial stenosis was detected in 98 of 309 patients. At least one vessel segment with significant stenosis was detected in 42/152 (27.6%), 34/80 (42.5%), 20/62 (32.3%), and 2/13 patients (15.4%) among the appropriate, uncertain, inappropriate, and not classifiable groups, respectively (Table 4). There was a significant difference in the frequency of >50% coronary stenosis among the appropriate, uncertain, and inappropriate rating groups (P = 0.03), and it was significantly higher in uncertain group than appropriate group. In contrast, the total number of coronary segments with >50% stenosis was not significantly different among the groups (P = 0.13; Table 5).

**Table 4:** **Significant CAD among 309 patients referred for cardiac CT**

**2010 AUC**	**No lesion [N (%)]**	**At least one vessel with >50% stenosis [N (%)]**	**Unable to evaluate [N (%)]**
Appropriate (N = 154)	95 (30.7%)	42 (13.6%)	17 (5.5%)
Uncertain (N = 80)	34 (11.0%)	34 (11.0%)	10 (3.3%)
Inappropriate (N = 62)	33 (10.7%)	20 (6.4%)	11 (3.6%)
Not classifiable (N = 13)	10 (3.3%)	2 (0.6%)	1 (0.3%)

Note _ AUC, appropriate use criteria; CAD, coronary artery disease.

**Table 5:** **Number of coronary segments with significant stenosis among patients with significant coronary arterial stenosis**

**2010 AUC**	**Number of coronary segments with >50% stenosis**		
	**1 segment**	**2 segments**	**3 or more segments**
Appropriate (N = 42)	22 (22.5%)	7 (7.1%)	13 (13.3%)
Uncertain (N = 34)	16 (16.3%)	5 (5.1%)	13 (13.3%)
Inappropriate (N = 20)	14 (14.3%)	2 (2.0%)	4 (4.1%)
Not classifiable (N = 2)	0	0	2 (2.0%)

Note _ AUC, appropriate use criteria.

## Discussion

This single-academic-center study presents the first attempt to evaluate the applicability of the 2010 AUC to the second-generation 320-row CT in clinical practice. We found that the proportion of uncertain (26%) rating was higher than that in previous studies (3% - 16%) (Cullen et al. 2013; Wasfy et al. 2012; Mazimba et al. 2012). One of the major reasons was the increase in CT examinations for detection of CAD in asymptomatic patients. The popularity of cardiac CT to screen patients with carotid artery stenosis and peripheral artery disease is increasing because these patients usually have multiple risk factors, including atherosclerosis. Among patients undergoing carotid endarterectomy or carotid artery stenting, 30%–50% of those with carotid artery stenosis had significant CAD (Shimada et al. 2005; Enomoto et al. 2013), and the severity of carotid artery stenosis and the extent of CAD were significantly correlated (Steinvil et al. 2011). In our survey, 43.5% of asymptomatic patients undergoing cardiac CT for preoperative evaluation of non-cardiac surgery had significant coronary artery stenosis. This might explain why the presence of significant CAD was higher in the uncertain rating group than in the appropriate rating group. We believe that non-invasive assessment of coexisting advanced atherosclerosis and CAD using current CT technology should be clinically important and appropriate for reducing perioperative myocardial infarction and stroke. The American College of Cardiology Foundation recognizes the importance of revising the criteria in timely manner in order to provide the cardiovascular community with the accurate indications (Taylor et al. 2010). Many uncertain-rating indications under the 2006 AUC changed to appropriate-rating under the 2010 AUC (Rich et al. 2012). Some of current uncertain-rating scenarios may potentially change to appropriate rating in the near future according to the rapid advances in CT technology.

Compared with invasive coronary angiography, cardiac CT using 64-row CT shows high diagnostic accuracy for detecting significant CAD (Sun et al. 2008). However, the widespread use of cardiac CT raises concerns regarding radiation exposure. With retrospective ECG-gated 64-row CT, the mean effective radiation dose is approximately 15 mSv (Alkadhi and Leschka 2011). While the radiation dose can be reduced by more than 50% with the step-and-shoot mode, this technique can only be used in patients strictly defined by their heart rate, heart rate variations, and body habitus. In our study, 241 of 309 (78%) patients underwent 1-beat prospective axial scan with iterative reconstruction technique, and their mean effective dose was only 1.8 mSv. In addition, we used 100- and 80-kVp scanning in 60% and 8% of patients, respectively. Low-voltage (80–100 kVp) techniques drastically reduce the radiation dose and effectively increase vascular enhancement (Hoffmann et al. 2012). Cardiac CT imaging plus iterative reconstruction reduces the radiation dose by 55% while yielding a contrast-to-noise ratio equal to 120-kVp CT imaging with the filtered back projection reconstruction (Hoffmann et al. 2012; Hou et al. 2012). In our study population, the mean contrast material volume was around 40 mL by using low-voltage technique and iterative reconstruction. A previous study (Rich et al. 2012) reported that low-voltage (100 kVp) cardiac CT was performed in 313 of 1293 (26%) patients between 2010 and 2011. In the present study, we used low-voltage cardiac scanning in 57% of patients. We consider that the application of iterative reconstruction for the smaller body habitus of Asians might result in a higher percentage of low-voltage CT. A previous study (Layritz et al. 2013) suggested that an automated attenuation-based selection of tube voltage and tube current can be used in clinical cardiac CT. We postulate that the appropriate selection of scan parameters with iterative reconstruction technique can increase the percentage of low-voltage CT, and reduce radiation exposure even in heavier patients in Western countries. Under these conditions, less-invasive cardiac CT would become more readily available, safe, and beneficial for patients with possible CAD, resulting in increase of clinical indications and appropriateness.

Our study has some limitations. First, we counted >50% stenosis as significant CAD on CT, but did not compare the CT finding with myocardial perfusion imaging. Stenosis of >50% does not directly mean myocardial ischemia. Also, we did not assess the net reclassification index of each appropriateness category. The proportion of obstructive CAD changes by the pre-test risk. Therefore, the assessment of the ability of cardiac CT to reclassify the risk of obstructive CAD should be important. Further studies should be performed to validate the relationship between uncertain category of the 2010 AUC and obstructive CAD. Second, our single-center study population was relatively small. Third, we did not compare the AUC rating between 64-row- and the updated 320-row machines. We posit that the differences between the previous study and our results might reflect our less-invasive cardiac CT influenced the clinical practice in the management of CAD patients. Fourth, we did not assess the interobserver agreement of the 2010 AUC ratings. Nonetheless, the ratings were based on consensus agreement between two experienced radiologists, and a very good interobserver agreement has been reported for this type of evaluation. Fifth, we did not compare image quality between the second- and first-generation 320-row CT. We believe that the updated 320-row CT provides better image quality due to a reduction in motion artifacts. Lastly, our interpretation of the cardiac CT images was not blinded to patient information. However, appropriateness ratings were determined before image interpretation and reporting.

## Conclusion

Cardiac CT with the second-generation 320-row CT may influence patient selection, leading to fewer indications that could not be classified, but a substantial increase in the number of uncertain scans. According to the 2010 AUC, uncertain rating is associated with a high prevalence of CAD. Our study may suggest that clinical indications of cardiac CT may become wider with the advances of the CT scanner and technology, resulting in early detection of significant CAD.
